# Metabolomics and Lipidomics Reveal the Metabolic Disorders Induced by Single and Combined Exposure of Fusarium Mycotoxins in IEC-6 Cells

**DOI:** 10.3390/foods14020230

**Published:** 2025-01-13

**Authors:** Xinlu Wang, Yanyang Xu, Haiqi Yu, Yushun Lu, Yongzhong Qian, Meng Wang

**Affiliations:** 1 Institute of Quality Standard and Testing Technology, Beijing Academy of Agriculture and Forestry Sciences, Beijing 100097, China; wangxinlu666@126.com (X.W.); yuhaiqi1015@163.com (H.Y.); 2 Institute of Quality Standards and Testing Technology for Agro-Products, Chinese Academy of Agricultural Sciences, Key Laboratory of Agri-Food Quality and Safety, Ministry of Agriculture and Rural Affairs, Beijing 100081, China; xuyanyang@caas.cn (Y.X.); luyushun@caas.cn (Y.L.)

**Keywords:** fusarium mycotoxins, combined toxicity, IEC-6 cells, metabolomics, lipidomics

## Abstract

Deoxynivalenol (DON), fumonisin B_1_ (FB_1_), and zearalenone (ZEN) are typical fusarium mycotoxins that occur worldwide in foodstuffs, posing significant health hazards to humans and animals. Single and combined exposure of DON, FB_1_, and ZEN leads to intestinal toxicity but the toxicology mechanism research is still limited. In this study, we explored the cytotoxicity effects of DON, FB_1_, ZEN, and their combination in rat intestinal epithelial cell line 6 (IEC-6) cells. Cell viability results showed that the cytotoxicity potency ranking was DON > ZEN > FB_1_. Furthermore, both DON + FB_1_ and DON + ZEN presented synergism to antagonism effects based on a combination index (CI)-isobologram equation model. Integrated metabolomics and lipidomics was adopted to explore cell metabolism disorders induced by fusarium mycotoxin exposure. A total of 2011 metabolites and 670 lipids were identified. An overlap of 37 and 62 differential compounds was confirmed after single and combined mycotoxin exposure by multivariate analysis, respectively. Some of the differential compounds were endocellular antioxidants and were significantly downregulated in mycotoxin exposure groups, indicating metabolic disorders as well as antioxidant capacity damage in cells. Pathway enrichment analysis annotated ethanol metabolism production of ROS by CYP2E1 was mainly involved in the disturbance of DON, FB_1_, and ZEN. The results obtained in this study help to define the toxicity effects of DON, FB_1_, and ZEN singly and in co-existence, providing an important scientific basis for combined risk recognition of mycotoxin contamination.

## 1. Introduction

Globally, mycotoxins are ubiquitously present in agricultural commodities and animal feeds [1]. Annually, up to 25% of food crops were affected by various mycotoxins, leading to the loss of nearly one billion tons of food and food products [2]. More than 400 mycotoxins have been confirmed worldwide in grains and other crops. Among them, deoxynivalenol (DON), fumonisin B_1_ (FB_1_), and zearalenone (ZEN) are of great significance [3]. The specific *Fusarium* species are the primary producers of these mycotoxins. For example, DON was mainly produced by *F. graminearum*, *F. nivale,* and *F. moniliforme*. FB_1_ was mainly produced by *F. moniliforme*, *F. verticillioides*, and *F. proliferatum*. ZEN was mainly produced by *F. oxysporum* and *F. graminearum*. According to a recent study of 300 samples collected from supermarkets and farms in Shanghai city of China, the detection rate of DON was the highest in soybean samples (75.64%) and wheat flour samples (86.67%), while the detection rate of FB_1_ was the highest in maize samples (98.00%) [4]. Similarly, previous studies reported that DON and its metabolites can be frequently detected in wheat samples and urinary samples of pregnant Croatian women [5,6]. ZEN is frequently found contaminating oats, corn, rice, barley, rye, and wheat in Europe and North America. Moreover, the incidence of ZEN was 61% in the emerging food of plant-based meat alternatives, indicating the severity of ZEN contamination [7]. In addition, co-contaminations of DON, FB_1_, and ZEN frequently occurred worldwide. It has been reported that 32% of cereal samples presented the co-occurrence of different mycotoxins, of which the co-occurrence of DON and ZEN was the prevalent combination [8]. Another study based on 576 maize and maize-derived food samples showed that the most frequent combination mycotoxins were DON and ZEN (52%), DON and FB_1_ (47%), and FB_1_ and ZEN (40%), respectively [9].

The intestinal epithelium is the primary site of mycotoxin exposure and has gained much attention when studying the intestinal toxicity of DON, FB_1_, and ZEN in recent years. DON is classified as a category three carcinogen by the International Agency for Research on Cancer [10]. DON exposure induced vomiting, weight loss, food refusal, and digestive disorders in animals [11]. FB_1_ is classified as a class 2B carcinogen. Previous studies showed that FB_1_ exposure decreased intestinal tight junction proteins in pigs and induced intestinal inflammatory injury in intestinal epithelial cells [12,13]. The toxicity of ZEN is known as an estrogen receptor activator. A recent study revealed that a peptide fragment of apolipoprotein E (ApoE) downregulation plays an important role in ZEN-induced intestinal immunotoxicity [14]. Moreover, the co-occurrence of fusarium mycotoxins exerts complex biological effects and has received increased research attention. For example, a previous study found that ZEN may have a synergistic effect on enhancing the activator protein (AP)-1 activity of the toxicity pathway of DON [15]. A recent study showed that DON and FB_1_ exhibited a synergistic or additive effect in facilitating intestinal inflammation [16]. However, it is not enough to reveal the combined toxicity risks of fusarium mycotoxins by only identifying the types of combined toxicity. Further studies should be conducted to explore the toxicity mechanisms of fusarium mycotoxins’ joint exposure.

Cell metabolism plays a crucial role in maintaining normal life status and is gaining much attention in toxicology mechanism studies. Integration of metabolomics and lipidomics is a good strategy to cover the majority of the metabolites in cells and can be applied to reflect cell damage after toxicant exposure. DON exposure reduced triglycerides (TGs) content and induced basal lipogenesis and metabolic disturbance in 3T3-L1 Cells [17]. Similarly, data from a 25-day trial based on metabolomics illustrated that DON exposure disturbed the glycerophospholipid, tryptophan, and glucose metabolism in rabbits [18]. A recent study identified 263 lipids in C6 cells and screened out 57 differential lipids after FB_1_ exposure, indicating the cytotoxic effects of FB_1_ [19]. Dai et.al revealed the endocrine disruptor effects of ZEN on female mice by untargeted lipidomics and found that the most prominent increase impacted by ZEN exposure concerned the diacylglycerols (DAG) and the phosphatidylglycerols (PG) [20]. However, there are few studies focused on the metabolic disorders induced by DON, FB_1_, and ZEN based on metabolomics and lipidomics simultaneously, especially for intestinal toxicity.

In the present study, cytotoxicity of DON, FB_1_, and ZEN singly and in combination was investigated in rat intestinal epithelial cell line 6 (IEC-6) cells. Moreover, integrated metabolomics and lipidomics was adopted to explore cell metabolism disorders induced by these three fusarium mycotoxins. To our knowledge, this work is the first to evaluate the intestinal cytotoxicity of DON, FB_1_, and ZEN using multi-omics techniques synchronously. The obtained results will provide new insights into the evaluation of the environmental risk and toxic mechanisms for fusarium mycotoxins.

## 2. Materials and Methods

### 2.1. Materials and Reagents

Deoxynivalenol (DON, purity > 99%), fumonisin B_1_ (FB_1_, purity ≥ 98%), and zearalenone (ZEN, purity > 99%) standards were purchased from MEIZHENG Bio-Tech (China). DON storage solution was dissolved in H_2_O. FB_1_ storage solution was dissolved in methanol/H_2_O (1:1, *v*/*v*). ZEN storage solution was dissolved in dimethyl sulfoxide (DMSO) (Sigma-Aldrich, St. Louis, MO, USA). The final concentrations of methanol and DMSO in medium were never higher than 0.1%.

Deionized water (18.2 MΩ) used for all experiments was purified with a Milli-Q system (Millipore, Boston, MA, USA). Acetonitrile (ACN), methanol, and 2-propanol (MERCK, Darmstadt, Germany) were high-performance liquid chromatography-mass spectrometry (HPLC-MS) grade. Ammonium acetate and formic acid were HPLC-MS grade (Sigma-Aldrich, St. Louis, MO, USA). Dichloromethane (MREDA, Beijing, China) was HPLC grade.

### 2.2. Cell Culture

IEC-6 cells (purchased from BNBIO Biotechnology Research Institute, Xinyang, China) were cultured in DMEM high glucose medium (Thermo Fisher Scientific, Boston, MA, USA) containing 10% (*v*/*v*) fetal bovine serum (Tianhang Biotechnology Co., Ltd., Meizhou, China), 100 U mL^−1^ penicillin, and 100 µg mL^−1^ streptomycin (Gibco, Boston, MA, USA) at 37 °C under 5% CO_2_ (Memmert CO_2_ Incubator, Schwabach, Germany). The IEC-6 cells were passaged when they reached 80% confluence.

### 2.3. Cell Viability Assay

IEC-6 cells were seeded into 96-well plates (3 × 10^4^ cells mL^−1^) for 24 h before mycotoxin exposure. Then, the culture medium was removed and replaced by the medium containing DON, FB_1_, and ZEN, respectively. The concentrations of DON ranged from 0.1 µM to 200 µM. The concentrations of FB_1_ ranged from 25 µM to 1000 µM. The concentrations of ZEN ranged from 20 µM to 500 µM. These concentrations were chosen according to a series of preliminary experiments. The cells treated with fresh medium were used as the control group. After exposure of 24 h, cell viability was detected using the Cell Counting Kit-8 (CCK-8, Dojindo Laboratories, Kumamoto, Japan) following the manufacturer’s instructions. After another incubation for 3 h, the optical density of each well was measured at 450 nm to determine the cell viability using a microplate reader (PerkinElmer, Waltham, MA, USA). Each condition was prepared with six replicates, and three independent 96-well plates were performed in different cell preparations. The exposure concentrations inducing 50% maximal inhibition (IC_50_) were obtained from dose–response curves by using the Origin 2018 software (OriginLab, Northampton, MA, USA).

### 2.4. Experimental Design of Mycotoxin Mixtures

Based on the measured individual IC_50_ values of DON, FB_1_, and ZEN, cells were treated with serial dilutions of each mycotoxin with an equitoxic constant mixture ratio. After a combined exposure of 24 h, cell viability was determined using CCK-8 kits. Then, the concentration addition (CA) model, independent action (IA) model, and combination index (CI)-isobologram equation model were applied to analyze the combined effects (DON + FB_1_, DON + ZEN) on cell viability inhibition. After that, the prediction results of different models were compared with the obtained experimental results to confirm the best prediction model.

To further explore the single and combined toxicity effects of mycotoxins on cell metabolism, the dosages of IC_25_ were chosen as exposure concentrations in the following non-targeted metabolomics and lipidomics study.

### 2.5. Sample Collection and Preparation for Non-Targeted Metabolomics and Lipidomics

After 24 h of exposure to different mycotoxins, cells were collected and prepared for metabolomics and lipidomics analysis. The preparation process was referred to in our previous study [21]. Briefly, cells were collected from each group and adjusted to the same density of 5 × 10^5^ cells mL^−1^. Afterward, cell membranes were lysed on ice by using an ultrasonic cell pulverizer (Xiaomei, Kunshan, China).

For metabolites extraction, 300 µL ice pre-chilled methanol was added to 100 µL cytosol to quench the enzymatic reactions. The mixture was then vortexed for 1 min and sonicated for 20 min in an ice bath. Next, the suspension was centrifugated at 10,000 r min^−1^ for 10 min. The supernatants were collected gently and filtered through 0.2 µm polytetrafluoroethylene filters to obtain metabolites for the analysis. The quality control (QC) samples were prepared using equal pooling of cell samples in all of the groups to monitor data stability and robustness.

For lipids extraction, 300 µL methanol–dichloromethane (2:1, *v*/*v*) was added to 100 µL of cytosol. The mixture was vortexed for 30 s by using an IKAMS3 basic vortex mixer (Germany) and centrifuged at 10,000 r min^−1^ for 5 min. Next, the supernatant was transferred to a glass tube and the extraction process was repeated once to improve the extraction efficiency. After that, the extracts were combined and evaporated for dryness using a nitrogen-blowing device and resuspended in 0.5 mL of methanol for lipid analysis. The QC samples were prepared using equal pooling of cell samples in all of the groups to monitor data stability and robustness.

### 2.6. UPLC-Xevo G2-S QTof MS Analysis of Cell Metabolites and Lipids

The Xevo G2-S QTof MS system (Waters, Milford, MA, USA), along with a Waters ACQUITY UPLC (Milford, MA, USA) system was used for sample analysis.

For non-targeted metabolomics analysis, chromatographic separation was performed on an ACQUITY UPLC HSS T3 column (2.1 mm × 100 mm × 1.8 µm, Waters) at 40 °C. The mobile phase A and B were ACN-containing 0.1% formic acid and water-containing 0.1% formic acid, respectively. The flow rate was 0.4 mL min^−1^. The gradient conditions were set as follows: 2% A for 0–0.5 min; 2–85% A for 0.5–3 min; 85–98% A for 3–7 min; 98% A for 7–11 min; 98–2% A for 11–11.5 min; 2% A for 11.5–16 min. The injection volume was 2 µL, and the sample tray was set at 4 °C. The QTof analysis was conducted in both positive and negative ion modes. The capillary voltage was set to 2.0 kV for positive ion mode and 1.0 kV for negative ion mode. The sampling cone voltages were set at 20.0 kV for both modes. Source temperature was set at 115 °C and desolvation temperature was set at 40 °C for both modes. MS data were acquired in Continuum MSE mode. Tof mass ranges from 50 to 1000 Da. A locked spray interface with a flow rate of 10 μL/min was utilized and locked leucine enkephalin mass at 200 pg/μL was employed to monitor the positive/negative ion patterns ( 556.2771 in ESI^+^/554.2615 in ESI^−^) and to record the accurate masses.

For non-targeted lipidomics analysis, chromatographic separation was performed on an Xbridge C18 column (2.1 mm × 100 mm × 3.5 µm, Waters) at 40 °C. The mobile phase A was ACN/2-propanol (1:9, *v*/*v*) with 10 mM ammonium formate and 0.1% formic acid. The mobile phase B was ACN/H_2_O (6:4, *v*/*v*) with 10 mM ammonium formate and 0.1% formic acid. The flow rate was 0.4 mL min^−1^. The gradient conditions were set as follows: 40% A for 0–2 min; 40–50% A for 2–3 min; 50–60% A for 3–10 min; 60–70% A for 10–12.5 min; 70–92% A for 12.5–17 min; 92–40% A for 17–17.5 min; 40% A for 17.5–20 min. The injection volume was 2 µL, and the sample tray was set at 4 °C. The QTof analysis was conducted in positive ion mode. The capillary voltage was set to 2.0 kV. The sampling cone voltages were set at 20.0 kV. The source temperature was set at 115 °C and the desolvation temperature was set at 40 °C. MS data were acquired in Continuum MSE mode. Tof mass ranges from 50 to 1000 Da. A locked spray interface with a flow rate of 10 μL/min was utilized and locked leucine enkephalin mass at 200 pg/μL was employed to monitor the positive ion pattern ([M + H] + 556.2771) and to record the accurate masses.

During instrumental analysis for both metabolomics and lipidomics, one QC sample was inserted between every eight real samples and then analyzed.

### 2.7. Data Processing and Statistical Analysis

Statistical analysis of changes in cell viability compared with the control was performed by using SPSS 21.0 (IBM, Almonk, NY, USA), with the threshold of *t*-test set at *p* < 0.05. The initially processed UPLC-QTof MS data were analyzed using Progenesis Qi V2.0 software (Waters, Milford, MA, USA). Multivariate data analysis was conducted with SIMCA-P 14.1 software (Umetrics, Malmo, Sweden). Heat maps were constructed using Multi Experiment Viewer software. Venn plot analysis was performed using the jvenn website (https://www.bioinformatics.com.cn/, accessed date 15 October 2024). The results of metabolomics pathway analysis were analyzed by MetaboAnalyst (https://www.metaboanalyst.ca/, accessed date 15 October 2024).

## 3. Results

### 3.1. Cytotoxicity Evaluation of Individual Exposure to DON, FB_1_ and ZEN

**Figure 1:**
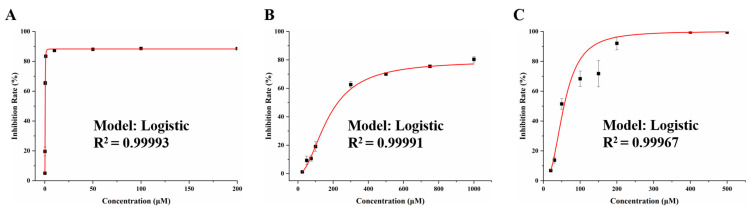
Non-linear fitting curves for the cytotoxic effects of DON (**A**), FB_1_ (**B**), ZEN (**C**).

CCK-8 assays were conducted to investigate the cytotoxicity effects of DON, FB_1_, and ZEN after 24 h exposure to IEC-6 cells. As shown in Figure 1, all of the three mycotoxins reduced cell viability in a concentration-dependent manner. According to the calculated IC_50_ values by Origin 2018 software, DON was the most cytotoxic on IEC-6 cells, followed by ZEN and FB_1_. The corresponding IC_50_ values of DON, ZEN, and FB_1_ were 0.37 μM, 55.97 μM, and 226.76 μM, respectively.

### 3.2. Combined Toxicity of DON-FB_1_ and DON-ZEN Mixtures

**Figure 2:**
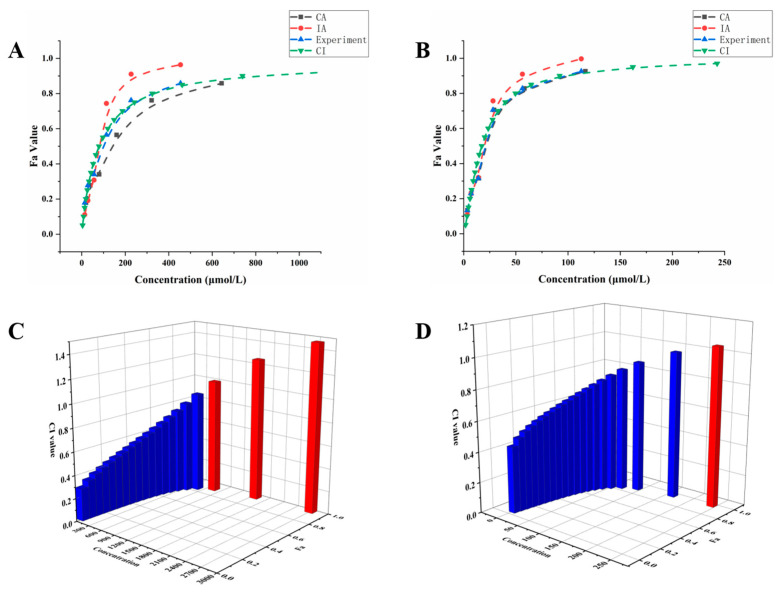
Prediction of combined toxicity modes of action based on CA, IA, and CI models for DON + FB_1_ (**A**) and DON + ZEN (**B**); the CI value of DON + FB_1_ (**C**) and DON + ZEN (**D**).

Based on the IC_50_ values of DON, FB_1_, and ZEN, a series of exposure concentrations from 1/8 IC_50_ to 4 IC_50_ were selected to investigate the combined toxicity of DON + FB_1_ and DON + ZEN on IEC-6 cells. The combined toxicity effects were evaluated by CA, IA, and CI models, respectively. As can be seen from Figure 2A,B, the fitting curves obtained by the CI model were closest to the data obtained from the actual experiments among the three models. Therefore, the CI model was used to analyze the combined toxicity effects in the following study. CI value was calculated using CompuSyn software, which provides a quantitative result to reveal the cytotoxic interactions. As shown in Figure 2C,D, the CI value of DON + FB_1_ ranged from 0.278 to 1.465, and the CI value of DON + ZEN ranged from 0.436 to 1.053. These results demonstrated that both DON + FB_1_ and DON + ZEN presented synergism effects at low exposure concentrations but antagonism effects at high exposure concentrations.

### 3.3. Multivariate Analysis of Metabolic Profiles in IEC-6 Cells After Mycotoxin Exposure

**Figure 3:**
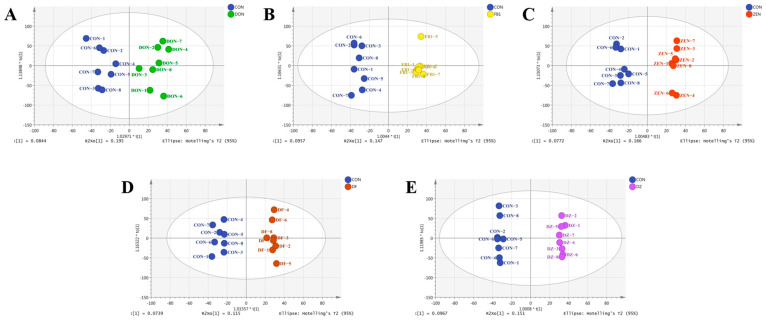
The OPLS-DA results of non-targeted metabolomics and lipidomics among the control group and different exposure groups including the DON group (**A**), the FB_1_ group (**B**), the ZEN group (**C**), the DON + FB_1_ group (**D**), and the DON + ZEN group (**E**), respectively.

The representative total ion current (TIC) of the QC sample acquired by metabolomics and lipidomics is shown in Figure S1. There were 7406 and 7520 compound ions recognized in the analysis of metabolomics and lipidomics by Progenesis Qi V2.0 software. A total of 2011 metabolites and 670 lipids were identified using the HMDB database and LIPIDMAPS database, respectively. Orthogonal partial latent structures-discriminant analysis (OPLS-DA) was conducted between the control group and different exposure groups by Simca 14.1 software. As shown in Figure 3, each dot represented the detected compound information of a sample, and all data points were distributed within the 95% confidence interval, indicating that none of the samples contained outliers. Moreover, the control group can be clearly distinguished from all of the mycotoxin exposure groups, illustrating the metabolic disorder in IEC-6 cells induced by mycotoxin exposure in single and binary conditions. The validation of the established OPLS-DA models was conducted using 200 permutation tests (Figure S2). R^2^ intercept ranged from 0.926 to 0.997, and Q^2^ intercept ranged from −0.099 to 0.083, indicating that the established OPLS-DA models were valid and not overfitting.

### 3.4. Potential Biomarkers Identification After Mycotoxin Exposure

**Figure 4:**
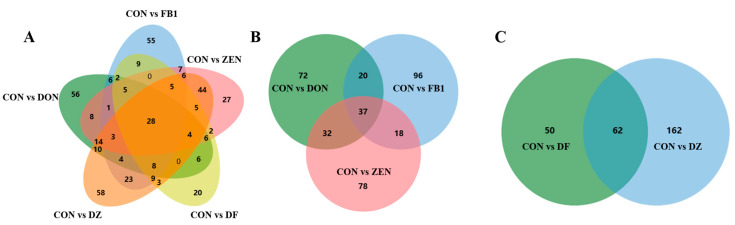
The Venn plots of the metabolites and lipids that undergone significant regulations in all of the groups (**A**); the single mycotoxin exposure groups (**B**); the binary mycotoxin exposure groups (**C**).

**Figure 5:**
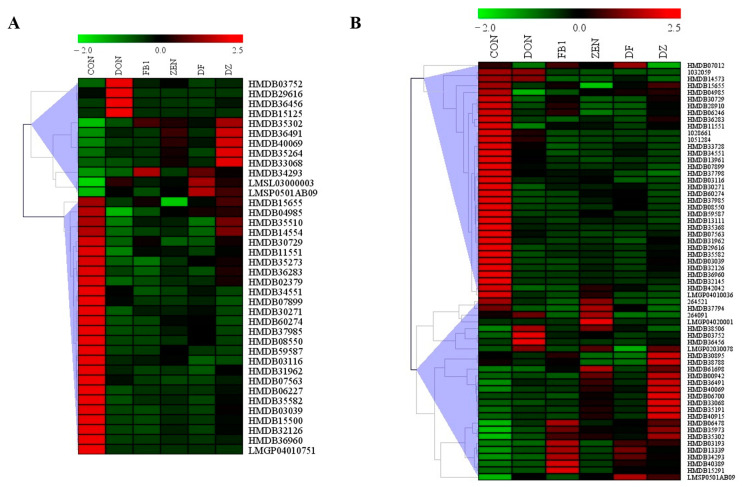
Heatmaps generated by the differential metabolites and lipids screened out from single mycotoxin exposure (**A**) and binary mycotoxin exposure (**B**).

To further investigate the disturbance induced by fusarium mycotoxin exposure, differential metabolites, and lipids were screened out based on the principles of *p* value < 0.05 and Fold Change (FC) value ≥ 2 or ≤0.5. As can be seen from Figure 4A, compared to the control group, there were 161, 171, 165, 112, and 224 differential metabolites and lipids, changing dramatically after exposure to DON, FB_1_, ZEN, DON + FB_1_, and DON + ZEN, respectively. Taking a further look at the differential compounds induced by single mycotoxin exposure (Figure 4B), an overlap of 37 compounds was found. Among these compounds, 10 were upregulated and 25 were downregulated significantly in all of the exposure groups. However, the metabolite of diazenedicarboxamide (ID: HMDB29616) and the lipid of 10-hydroxy-3-methoxy-1,3,5,7-cadinatetraen-9-one (ID: HMDB36456) presented different changing trends in different exposure groups. Correspondingly, Figure 4C showed that a total of 62 compounds co-occurred after binary mycotoxin exposure (DON + FB_1_ and DON + ZEN). The results demonstrated that co-occurrence of fusarium mycotoxins caused more severe metabolic disorders than single fusarium mycotoxin exposure. Among all of the differential metabolites and lipids screened out from the binary mycotoxin exposure, a total of 35 compounds downregulated and 18 compounds upregulated, respectively. There were nine compounds that presented complex (either upregulated or downregulated) changing trends in different exposure groups. It is noteworthy that among the 28 differential compounds repeatedly involved in single exposure and binary exposure (Figure 4A), seven of them are lipids. They are LysoPC(10:0/0:0), DG(20:5(5Z,8Z,11Z,14Z,17Z)/16:1(9Z)/0:0), PC(14:1(9Z)/14:0), 10-Hydroxy-3-methoxy-1,3,5,7-cadinatetraen-9-one, PC(22:0/22:5(7Z,10Z,13Z,16Z,19Z)), MG(0:0/22:0/0:0) and LacCer(d18:1/24:1(15Z)). As can be seen from Figure 5, the proportion of downregulated compounds was higher than that of upregulated compounds. This illustrated that fusarium mycotoxin exposure dominated metabolite synthesis inhibition in IEC-6 cells. Detailed information on the 37 and 62 differential compounds is shown in Supplementary Materials S2 and Supplementary Materials S3, respectively.

### 3.5. Metabolic Pathway Analysis

**Figure 6:**
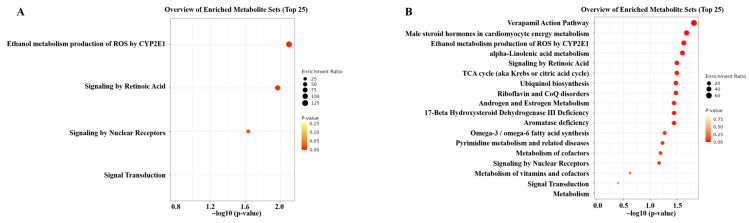
Pathway enrichment analysis based on the differential metabolites and lipids screened out from single mycotoxin exposure (**A**) and binary mycotoxin exposure (**B**).

Based on the differential metabolites and lipids screened out from the single and combined fusarium mycotoxin exposure groups, metabolic pathways analysis was conducted using the Metaboanalyst website. The enrichment analysis results showed that 4 metabolic pathways were affected after single exposure of DON, FB_1_, and ZEN (Figure 6A). Among them, 3 metabolic pathways were predicted to have a *p* value < 0.05. Correspondingly, there were 18 metabolic pathways disturbed after the binary exposure of DON + FB_1_ and DON + ZEN (Figure 6B). Among them, a total of 11 metabolic pathways were predicted to have a *p* value < 0.05. The results confirmed that combined fusarium mycotoxin exposure induced more severe metabolic disorders than single fusarium mycotoxin exposure. It is noteworthy that ethanol metabolism production of ROS by CYP2E1 was the most significantly disturbed pathway induced by single fusarium mycotoxin exposure. The verapamil action pathway, male steroid hormones in cardiomyocyte energy metabolism, and ethanol metabolism production of ROS by CYP2E1 were the top three pathways disturbed after DON + FB_1_ and DON + ZEN exposure.

## 4. Discussion

Cell models have been widely used in toxicology studies with advantages of high throughput and low cost compared with animal models. Numerous studies have investigated the cytotoxicity of different fusarium mycotoxins by using different cell models. A previous study explored the cytotoxicity of DON in the porcine jejunum epithelial cell line (IPEC-J2), and the results showed that 8 μM of DON exposure for 12 h significantly decreased the viability of IPEC-J2 cells (*p* < 0.01) [22]. It can be seen that the concentration of DON exposure to IPEC-J2 cells was much higher than that used on IEC-6 cells in the present study, demonstrating that different types of intestinal cells have various degrees of sensitivity to DON. According to the calculated IC_50_ values, we found the cytotoxicity potency ranking was DON > ZEN > FB_1_ in IEC-6 cells. Similar to our findings, it has been reported that DON was the most cytotoxic in IPEC-J2 cells, followed by ZEN and FB_1_ [23]. Previous studies have explored the IC_50_ values of ZEN in HepG2 cells and CHO-K1 cells, respectively [24,25]. However, there are limited studies exploring the IC_50_ values in intestinal cell models, and our study provided the IC_50_ value of ZEN in IEC-6 cells after 24 h exposure for the first time. Similarly, recent studies have revealed the intestinal inflammatory injury induced by FB_1_ exposure tested on IPEC-J2 cells [13,26], while the IC_50_ information on FB_1_ in intestinal cells is still scarce and our study provided the information first in IEC-6 cells.

Due to the most cytotoxicity of DON on IEC-6 cells, combined exposure of DON + FB_1_ and DON + ZEN was conducted in experiments to explore the binary cytotoxicity effects. Consistently, both DON + FB_1_ and DON + ZEN showed synergism effects at low exposure concentrations but antagonism effects at high exposure concentrations. It demonstrated that the combined toxicity effects of fusarium mycotoxins were complex. The complexity was consistent with a previous study, which found that a combination of ZEN and Aflatoxin B_1_ (AFB_1_) showed nearly additive effects at low concentrations and antagonism effects at high concentrations in Caco-2 cells [27]. As mentioned before, DON and FB_1_ exhibited a synergistic or additive effect in facilitating intestinal inflammation via pyroptosis in vivo and in vitro [16]. However, DON showed unexpected antagonism effects with emerging *Fusarium* mycotoxin enniatins (ENA, ENA_1_, ENB, and ENB_1_) in Caco-2 cells, particularly ENB at 1/4 IC_50_ [28]. In exploring the combined toxicity of FB_1_ and AFB_1_, synergistic effects were seen in Caco-2 cells [29]. Co-contamination of DON and ZEN presented an additive effect in Caco-2 cells and caused antagonistic toxicity in human colon carcinoma (HCT116) cells [30]. The combined cytotoxicity effects of fusarium mycotoxins were shown to vary depending on the cell used, the exposure concentrations and exposure time, and the type of damage analyzed [31]. Further studies should be carried out to explore the combined toxicity effect mechanisms, especially in the changes of mode of action (MOA).

Metabolomics and lipidomics are effective methods of exploring specific molecular mechanisms and have been widely applied in cytotoxicity effect studies. Unfortunately, there are limited studies revealing the single and combined cytotoxicity effects of fusarium mycotoxin exposure using metabolomics and lipidomics simultaneously. In this study, we explored the metabolic disorders caused by DON, FB_1_, and ZEN exposure by using metabolomics and lipidomics for the first time. A total of 2681 metabolites and lipids were identified. Furthermore, there were 37 and 62 intersecting differential compounds screened out in the single and combined exposure groups, respectively. To be specific, there were 25 and 35 compounds downregulated significantly in single and combined exposure groups. Correspondingly, there were 10 and 18 compounds upregulated significantly in single and combined exposure groups. Furthermore, there were 2 and 9 compounds that presented complex changing trends in single and combined exposure groups. It can be seen that most of the differential compounds showed obvious downregulation in fusarium mycotoxins exposure groups. Similar to our results, recent studies showed that DON exposure downregulated multi-amino metabolism and inhibited the cell cycle by using metabolomics [10,32]. Moreover, after 28 days of exposure to DON in mice, lipogenesis genes (*Dgat2*, *Srebp-1c*, *Pparγ*) downregulated significantly, indicating the lipid synthesis inhibition induced by DON exposure [33]. Our previous study focused on the neurotoxicity of FB_1_ on C6 cells by lipidomics analysis and found that there were 34 differential lipids decreased dramatically in the FB_1_ exposure group [19]. However, there has been no study investigating the metabolic disorder caused by FB_1_ exposure using metabolomics analysis. The present study provided new insights into FB_1_-induced toxicity effects and showed a similar changing trend to previous studies. Interestingly, some of the differential downregulated metabolites and lipids play vital roles in maintaining cell structures and functions. For example, gravelliferone, medicagenic, tsugaric acid B, tsugaric acid C, cyananin, and isoacitretin have beneficial effects on antioxidation. Antioxidants play important roles in maintaining health, preventing oxidative stress, and relieving inflammatory responses in cells. The decrease in these antioxidants demonstrated that fusarium mycotoxin exposure induced metabolic disorders as well as antioxidant capacity damage in cells. Coincidently, tsugaric acid C was screened out as a downregulation biomarker in exploring dextran sulfate sodium (DSS)-induced intestinal inflammation in ulcerative colitis (UC) mice, indicating the important role of tsugaric acid C in resisting intestinal inflammation [34]. Correspondingly, PC(22:0/22:5(7Z,10Z,13Z,16Z,19Z)), MG(0:0/22:0/0:0), and LacCer(d18:1/24:1(15Z)) play important roles in cell signaling transduction and cell membrane maintaining. A significant decrease in these lipids indicated the potential damage to the cell membrane. Moreover, the potential lipid biomarkers of PC(14:1(9Z)/14:0), PC(22:0/22:5(7Z,10Z,13Z,16Z,19Z)), PG(16:0/20:4), and PE(P-20:0/18:4(6Z,9Z,12Z,15Z)) belong to phospholipids, which are particularly susceptible to oxidative damage mediated by reactive oxygen species (ROS) due to their content of polyunsaturated fatty acid chains. Oxidative stress occurs when oxidation exceeds the antioxidant capacity. Previous studies have confirmed that fusarium mycotoxin exposure caused a significant accumulation of ROS, which is an end product of lipid peroxidation [3,19]. In other words, excessive ROS production initiates changes in the lipid layer composition of the cell membrane, accompanied by the lipid peroxidation process. ZEN is widely known to have estrogenic effects, and our study confirmed the estrogenic effects of ZEN by finding that two kinds of hormones (methyltestosterone and allylestrenol) were significantly disturbed after ZEN exposure in IEC-6 cells. Similarly, a previous study screened out epiestradiol as a kind of potential biomarker after ZEN exposure in ANA-1 cells by using metabolomics analysis [35].

Pathway enrichment analysis results showed that ethanol metabolism production of ROS by CYP2E1 was annotated based on the differential compounds screened out from both single and combined exposure with a *p* value < 0.05. The upregulated expression of CYP2E1 accelerates ethanol oxidation, leading to an accumulation of acetaldehyde and ROS. CYCP2E1 is an important target for exploring mycotoxin toxicity. A recent study revealed that DON exposure significantly increased the expression of CYP2E1 and the level of cell lipid ROS in hepatocytes. Moreover, the knockdown of CYP2E1 alleviated DON-induced high levels of lipid ROS and ferroptosis [36]. Similarly, aflatoxin G_1_ (AFG_1_) exposure upregulated CYP2E1 through the NF-κB pathway, causing oxidative DNA damage in the human gastric cell line GES-1 [37]. Published literature has indicated that CYP2E1 plays a regulatory role in lipid peroxidation and lipid metabolism [38,39]. This may explain the occurrence of differential lipids screened out after fusarium mycotoxin exposure in the present study. However, further studies should be conducted to explore the regulatory mechanism of CYP2E1 and ROS generation after single and combined exposure to fusarium mycotoxins. Overall, our present study based on metabolomics and lipidomics provides new insights for understanding the intestinal cytotoxicity of fusarium mycotoxins after single and combined exposure.

## 5. Conclusions

This study confirmed the cytotoxicity of DON, FB_1_, and ZEN in IEC-6 cells with the cytotoxicity potency ranking of DON > ZEN > FB_1_. The binary combination result of DON + FB_1_ and DON + ZEN showed synergism to antagonism effects based on CI analysis. Integrated metabolomics and lipidomics were applied for the first time to analyze the cell metabolism disorder induced by fusarium mycotoxin exposure. A total of 2011 metabolites and 670 lipids were identified using the HMDB database and the LIPIDMAPS database, respectively. An overlap of 37 and 62 differential compounds, which was confirmed with the principles of *p* < 0.05 and FC ≥ 2 or ≤0.5, was of particular concern. A majority of the differential metabolites and lipids presented significant downregulation trends in mycotoxin exposure groups, and some of them have antioxidant abilities. The decrease in these antioxidants demonstrated that fusarium mycotoxin exposure induced metabolic disorders as well as antioxidant capacity damage in cells. Pathway enrichment analysis focused on the ethanol metabolism production of ROS by CYP2E1, indicating the important role of CYCP2E1 in regulating the toxicity mechanisms of DON, FB_1_, and ZEN. The results provide new insights into the study of health disorders related to fusarium mycotoxin exposure by using cellular models.

## Data Availability

The datasets generated for this study are available on request to the corresponding author.

## Supplementary Materials

The following supporting information can be downloaded at: https://www.mdpi.com/article/10.3390/foods14020230/s1, Figure S1: The representative total ion chromatogram (TIC) of metabolomics in the positive ion mode (A); the representative total ion chromatogram (TIC) of metabolomics in the negative ion mode (B); the representative total ion chromatogram (TIC) of lipidomics in the positive ion mode (C); Figure S2: The permutation test results of non-targeted metabolomics and lipidomics among the control group and different exposure groups including the DON group (A), the FB1 group (B), the ZEN group (C), the DON + FB1 group (D), and the DON + ZEN group (E), respectively; Supplementary Materials S2: detailed information of the 37 overlap differential compounds screened out from single mycotoxin exposure groups; Supplementary Materials S3: detailed information of the 62 overlap differential compounds screened out from binary mycotoxin exposure groups.
