# Supplementary material for: Metabolomics and Lipidomics Reveal the Metabolic Disorders Induced by Single and Combined Exposure of Fusarium Mycotoxins in IEC-6 Cells
Source: Foods. 2025 Jan 13;14(2):230. doi: 10.3390/foods14020230 (PMC11765315; doi:10.3390/foods14020230)
Supplement: Supplementary file 1 [file foods-14-00230-s001.zip › Supplementary Materials/Supplementary Materials.pdf]

# Metabolomics and Lipidomics Reveal the Metabolic Disorders Induced by Single and Combined Exposure of Fusarium Mycotoxins in IEC-6 Cells

**Xinlu Wang [1], Yanyang Xu [2], Haiqi Yu [1], Yushun Lu [2], Yongzhong Qian [2,*] and Meng Wang [1,*]**

[1] Institute of Quality Standard and Testing Technology, Beijing Academy of Agriculture and Forestry Sciences, Beijing 100097, China; wangxinlu666@126.com (X.W.); yuhaiqi1015@163.com (H.Y.)
[2] Institute of Quality Standards and Testing Technology for Agro-Products, Chinese Academy of Agricultural Sciences, Key Laboratory of Agri-Food Quality and Safety, Ministry of Agriculture and Rural Affairs, Beijing 100081, China; xuyanyang@caas.cn (Y.X.); luyushun@caas.cn (Y.L.)
* Correspondence: qianyongzhong@caas.cn (Y.Q.); wangm@iqstt.cn (M.W.)

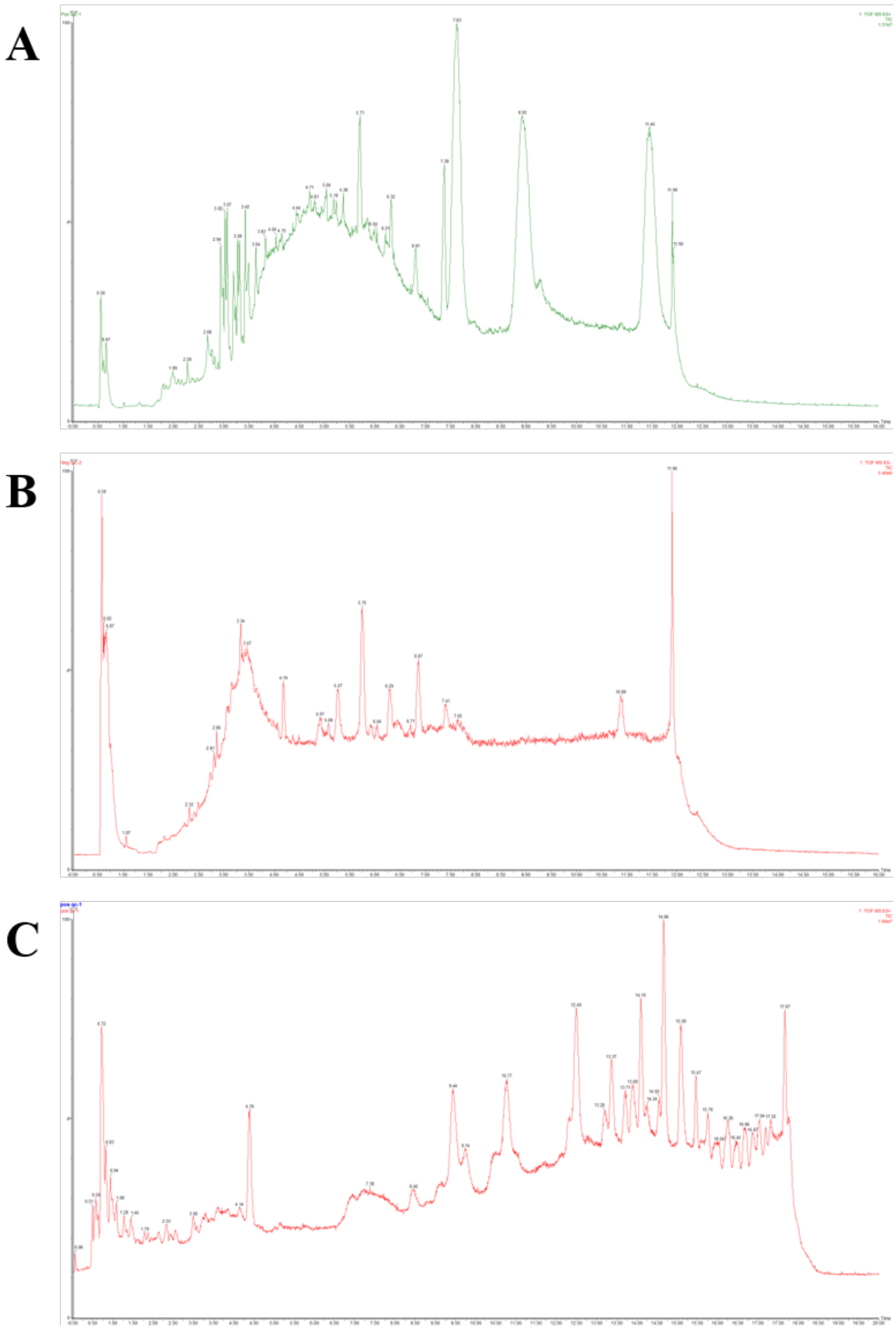


Figure S1. The representative total ion chromatogram (TIC) of metabolomics in the positive ion mode (A); the representative total ion chromatogram (TIC) of metabolomics in the negative ion mode (B); the representative total ion chromatogram (TIC) of lipidomics in the positive ion mode (C).

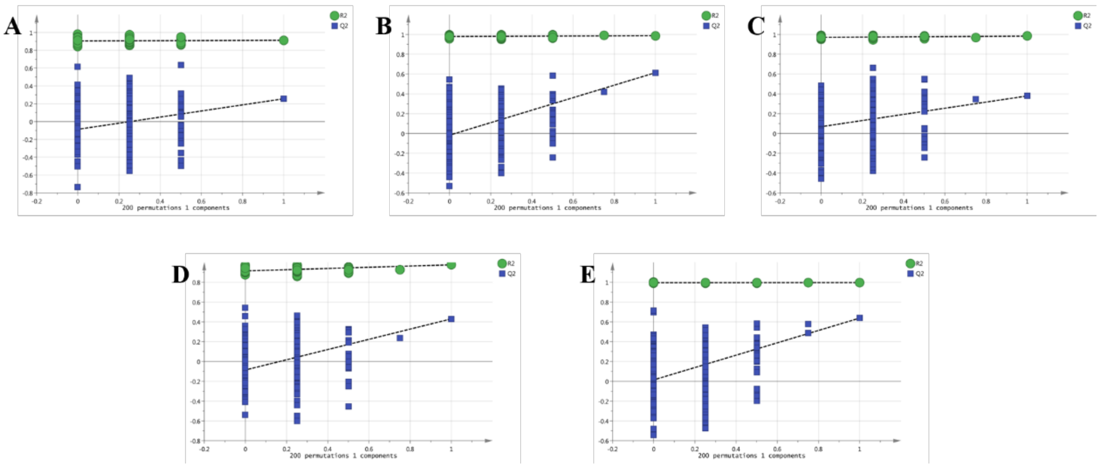


Figure S2. The permutation test results of non-targeted metabolomics and lipidomics among the control group and different exposure groups including the DON group (A), the FB1 group (B), the ZEN group (C), the DON + FB1 group (D), and the DON + ZEN group (E), respectively.
